# Pivoting injury prevention efforts during a pandemic: results of an international survey

**DOI:** 10.1186/s40621-023-00472-3

**Published:** 2023-11-16

**Authors:** Tanya Charyk Stewart, Purnima Unni, Holly Renee Hanson, Jason Gilliland, Andrew Clark, Douglas D. Fraser

**Affiliations:** 1 London Health Sciences Centre, London, ON Canada; 2 Department of Paediatrics, Schulich School of Medicine and Dentistry, Western University, London, ON Canada; 3 Department of Pathology and Laboratory Medicine, Schulich School of Medicine and Dentistry, Western University, London, ON Canada; 4 Lawson Health Research Institute, London, ON Canada; 5 Monroe Carell Jr. Children’s Hospital at Vanderbilt, Nashville, TN USA; 6 Department of Geography and Environment, Western University, London, ON Canada; 7 Department of Epidemiology and Biostatistics, Schulich School of Medicine and Dentistry, Western University, London, ON Canada; 8 School of Health Studies, Western University, London, ON Canada; 9 Children’s Health Research Institute, London, ON Canada; 10 School of Design, Fanshawe College, London, ON Canada

**Keywords:** Injury prevention, COVID-19, Pediatric trauma, Survey

## Abstract

**Background:**

The COVID-19 a pandemic changed the world. Public health directives to socially distance with stay-at-home orders altered injury risk factor exposure, resulting injury patterns and conducting injury prevention (IP). The objective of this study was to determine the impact the COVID-19 pandemic on injury and IP at North American trauma centers (TC).

**Results:**

Sixty-two responses were received from pediatric (44%), adult (11%), and combined (31%) TC, from 22 American states, 5 Canadian provinces and Australia. The majority (91%) of programs targeted age groups from birth to 15 years old. Nearly one-third reported IP to be less of an institutional priority with funding redistributed in 15% of centers [median (IQR) − 25% (− 43, 1)], and resultant staffing changes at 38% of centers. A decrease in IP efforts was reported at 64% of TC. Overall, the majority of respondents reviewed injury data, with the top reported increased mechanisms mainly intentional: Firearm-related (75%), assaults (72%), and abuse (71%). Leading increased unintentional injuries were injuries occurring in the home such as falls (70%), followed by ATV (62%), and cycling (57%). Sites pivoted by presenting (74%) or participating (73%) in IP education virtually, social media posts (61%) and the addition of technology (29%). Top barriers were redeployment of partners (45%) and staff (31%), as well as lack of technology (40%) in the target population. Facilitators were technology at TC (74%), support of trauma program (63%), and having IP funding maintained (55%).

**Conclusions:**

Nearly two-thirds of TC decreased IP efforts during the pandemic due to staffing and funding reductions. The leading reported increased injuries were intentional, indicating that violence prevention is needed, along with support for mental health. While TC successfully pivoted by using technology, access issues in the target population was a barrier resulting in health inequities.

**Supplementary Information:**

The online version contains supplementary material available at 10.1186/s40621-023-00472-3.

## Background

The declaration of the COVID-19 pandemic, as a result of the severe acute respiratory syndrome coronavirus 2 (SARS-CoV-2), by the World Health Organization (WHO) on March 11, 2020 changed the world (World Health Organization 2020). In response to the declaration, governments throughout the world implemented strict public health directives in an attempted to “flatten the curve”; effectively reducing infections and preventing further transmission of the virus (Gilmartin et al. 2022; Strassle et al. 2022; Teslya et al. 2020). Globally, lockdowns, or stay-at-home orders, were put in place to help people socially distance, resulting in the closure of non-essential business, school, playgrounds, recreation facilities and daycares. Across North America, large public gatherings were prohibited and organized sporting activities were put on hold. This upheaval in daily life for both adults and children fundamentally changed our exposure to injury risk factors (Gilmartin et al. 2022; Law et al. 2022; Sinyor et al. 2022).

As a result of the pandemic and associated public health restrictions, the patterns and mechanisms of injury sustained during this time were altered globally. The direction of the change was dependent on geographic location and population. Previous research in the United States and Canada initially found declines in visits to the emergency departments (ED) or trauma admissions, for injuries caused by motor vehicle collisions (MVC) (Law et al. 2022; Harmon et al. 2021; Keays et al. 2020; Hassan et al. 2020), sports-related injuries (Keays et al. 2020) and falls (Law et al. 2022; Harmon et al. 2021). Injuries related to other mechanisms were found to increase, including cycling injuries (Hanson and Pomerantz 2022) and penetrating injury from firearms and stabbings (Hanson and Pomerantz 2022; Ng et al. 2022; Collings et al. 2022). Assaults, child abuse and maltreatment, and self-harm had more mixed results on the direction of change, depending on the population and geographic region, increasing in some regions (Hassan et al. 2020; Hanson and Pomerantz 2022; Ng et al. 2022; Holland et al. 2021a), and decreasing (Kaiser et al. 2021) or no significant change in other regions (Law et al. 2022). What was not in question, and continues to influence injury patterns, especially those as a result of an intentional mechanism, was the impact of the pandemic on mental health. Increased mental health concerns was due to a complex interplay of factors including society fear, uncertainty about the virus, prolonged isolation due to social distancing, financial insecurity, increased substance use, as well as school closures and the move to online learning. These factors were particularly harmful for the mental health of children and adolescents (Sinyor et al. 2022; John et al. 2020; Gunnell et al. 2020; Godinic et al. 2020; Ho et al. 2020). This may affect suicidal and violent behavior, as well as substance use, which has the potential to impact the types of injuries, specifically intentional injuries, experienced during the pandemic. It highlights the need for mental health, substance use and violence risk screening and prevention efforts during these turbulent times (Holland et al. 2021a).

Along with the injury risk factors and patterns changing due to the pandemic and government mandated lockdowns, the approach used to mitigate these emerging injury types also needed to be altered. Typically, pre-pandemic, the majority of injury prevention (IP) initiatives and education were completed in-person and at public events. With the closure of schools and cessation of large public gatherings, the methods for conducting IP required a pivot to adapt to the new public health restrictions. Exactly how this was accomplished at adult and pediatric trauma centers throughout North America, along with other repercussions trauma centers faced as a result of the pandemic, has not been well documented. The objective of this study was to determine the impact the COVID-19 pandemic on injury and its prevention at trauma centers throughout the United States (US) and Canada.

## Methods

### Study population and sampling methods

This cross-sectional study on the impact of the COVID-19 pandemic on injury and IP practices was undertaken with members of the following trauma/IP associations: Pediatric Trauma Society (PTS), Injury Free Coalition for Kids® (IFCK) and the Trauma Association of Canada (TAC). These associations have members that are health care providers working in trauma centers throughout North America, as well as Australia due to the Australasian Trauma Society’s affiliation with TAC. As not all IP practitioners at trauma centers are members of these three associations, a snowball sampling strategy was also employed (Ruel et al. 2016), allowing recipients of the survey invitation to forward the survey link on to the IP coordinator or specialist at their institution, who may not be a member of one of the three participating associations. This allowed their institution's IP experience during the COVID-19 pandemic to be accurately represented.

### Questionnaire development and survey administration

A standardized approach for the design and conduct of survey for clinicians was followed (Burns et al. 2008). A questionnaire was designed utilizing the REDCap (Research Electronic Data Capture) platform, a web-based data capturing and survey tool that allows the secure storage and encryption of data (Harris et al. 2009). Our resulting survey is reported herein according to the Consensus-Based Checklist for Reporting of Survey Studies (CROSS) (Sharma et al. 2021). The questionnaire contained quantitative questions, including 7-point Likert scale questions (*extreme/moderate/slight increase* and *decrease*, or *remained the same*), as well as qualitative open-ended questions addressing the following survey domains: (1) IP initiatives; (2) injury data; (3) staffing; (4) IP funding and priority; (5) IP pandemic pivots; (6) facilitators and barriers; (7) education and training; and (8) demographics.

The survey underwent an expert-driven pre-test with IP experts from the US and Canada. This involved a pre-test survey assessing question wording, response categories, as well as identifying any additional questions that needed to be included to cover the comprehensiveness of the domains. This pre-test survey link was distributed by email through REDCap and completed by the IP experts, including the chairs or a representative of the IP committees of the participating trauma/IP associations. Following this pre-test survey, two online focus groups and debriefing assessments were conducted to finalize the questions and multiple choice responses (Ruel et al. 2016). Participants reviewed the survey, then suggested revisions to the questionnaire to improve clarity, readability and comprehensiveness. The survey process, format and any technologic issues, including survey access and branching logic, were also reviewed. Face and content validity of the questionnaire was also assessed during this pre-testing phase of questionnaire development. Questions were added or revised following input from the IP experts to improve the questionnaire and decrease the chances of the respondents misinterpreting questions (Burns et al. 2008). A copy of the final survey is presented in Additional file 1.

The online link for the final survey was distributed to all PTS, IFCK® and TAC members from September to October of 2021, with initial survey invitation and up to three reminders, or until the survey was completed, in a modified multiple-wave protocol of the Dillman tailored design technique for internet surveys (Dillman et al. 2014). While the survey link was sent to all members of PTS, IFCK® and TAC, not all members perform IP activities as part of their responsibilities, so the survey was not applicable to them. As well, given the snowball sampling methodology used (Ruel et al. 2016), members receiving the survey invitation could forward the survey link on to the IP coordinator or specialist at their institution, if there were not members of one of the associations, and did not receive the invitation initially. The Western University Health Sciences Research Ethics Board (HSREB) reviewed and approved this study (REB# 117944).

### Statistical analysis

Descriptive statistics including counts, percentage, median and interquartile (IQR) range were calculated using IBM® SPSS® Statistics Version 28.0.0 (IBM Corporation, Armonk, NY). Pearson chi-square test was used to compare proportions of mechanism of injury by country, US compared to Canada. Imputation was not used for any question with missing responses, as each IP practitioner’s practices and experience were unique to them, so imputing values based on other IP practitioners’ responses would not be valid. Theming analyses were performed on qualitative, open-ended questions.

## Results

### Respondents and demographics

**Fig. 1:**
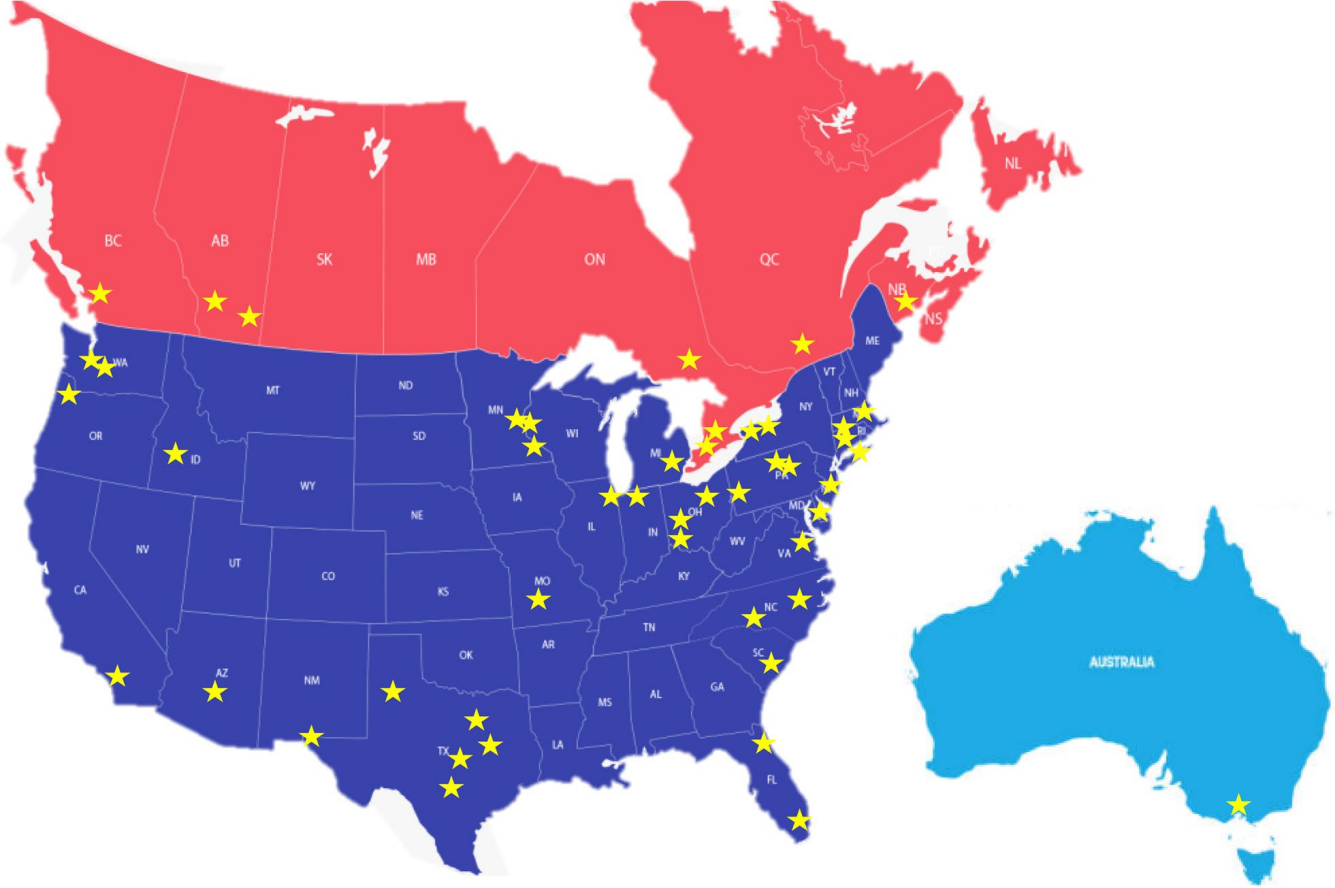
Geographic representation of responses (*n* = 62) from 22 American states, 5 Canadian provinces and Melbourne, Australia

There was a total of 62 responses to the online survey with geographic representation from 22 American states, 5 Canadian provinces and from Melbourne, Australia (Fig. 1). Due to the snowball sampling methodology used, an accurate survey response rate could not be calculated, as the denominator could not be determined. Based on the trauma center type, name and city, it was determined there were no duplication in responses from one trauma program, so all initial 62 responses remained in the results and represented unique trauma centers.

**Fig. 2:**
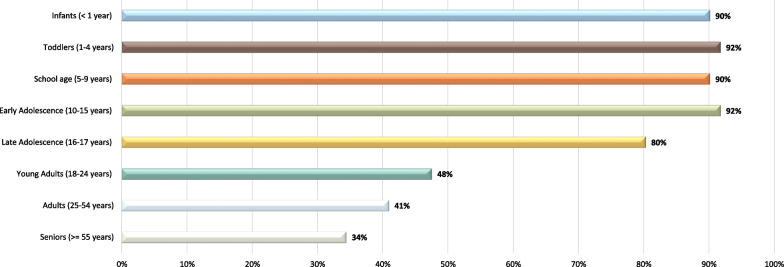
Age groups reported targeted for injury prevention efforts

IP specialist was the leading role of respondents (*n* = 39/62; 62.9%), followed by trauma medical director/physician (*n* = 7/62; 11.3%) and trauma program manager (*n* = 6/62; 9.7%), with the median years of IP experience being 11 years (IQR 5–20). Eighty-seven percent (*n* = 54/62) of respondents worked at a trauma center (*n* = 37/54 respondents; 68.5% Level I). Of those that worked in a trauma center, 51.9% (*n* = 28/54) were a pediatric trauma center, 35.2% (*n* = 19/54) a combined adult and pediatric trauma center and 13.0% (*n* = 7/54) an adult trauma center. The overwhelming majority of respondents target children and adolescents with their IP programs, with over 90% of respondents targeting infants, toddlers, school age and early adolescence age groups up to age 15 years (Fig. 2). In total, 29.5% (*n* = 18/61) of respondents include all age groups in their IP programing.

### Injury data and trends

**Fig. 3:**
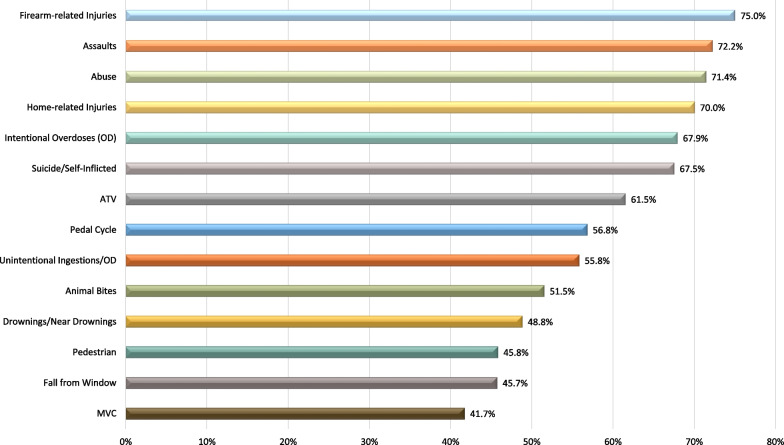
Reported increases in injury mechanisms following the declaration of the COVID-19 pandemic

The majority (*n* = 5/611; 83.6%) of respondents reviewed the data to keep up with the most current injury epidemiology and any changes to injury trends that occurred during to the pandemic. Most respondents that reviewed the data (*n* = 42/51; 82.4%) reviewed Trauma Registry Data. Approximately half also examined the emergency department (ED) data (*n* = 30/51; 58.8%), 45.1% (*n* = 23/51) reviewed in-patient data, with 29.4% (*n* = 15/51) reviewing the coroner’s death data. Figure 3 depicts the reported increases in injury mechanisms. Firearm-related injuries had the highest reported increased incidence during the pandemic at 75.0%, which could be either an intentional or unintentional injury. Four out of five (80%) of the next highest injury pattern increases were intentional mechanisms including assaults 72.2%; abuse 71.4%; intentional overdoses 67.9%; and suicide/self-inflicted injury 67.5%. A sub-analysis of mechanism of injury was undertaken by country comparing proportions for the US and Canada. A statistically significant difference in increase in firearm-related injuries was found by country, with 84.2% of American respondents reporting an increase in firearm-related injuries at their institutions, compared to 16.7% of Canadian respondents reporting this increase (*p* = 0.006). Statistically significant higher increases in abuse cases at American trauma centers (79.4% versus 28.6%; *p* = 0.014) and significant increases in unintentional overdoses reported at 80.0% of Canadian trauma centers compared to 46.7% of American trauma centers (*p* = 0.029).

### IP priority and funding

When asked, “Since the start of the pandemic, how do you feel your trauma center views IP in terms of an institutional priority?”, 42.6% (*n* = 26/61) of respondents that replied reported no change in the institution’s priority level of IP, 31.1% (*n* = 19/61) reported IP was less important of a priority in their institution and 23.0% (*n* = 14/61) reported it was a more important priority. Two respondents (*n*=2/61; 3.3%) reported they did not know. Of those that were aware of IP funding, 15.4% (*n* = 8/52) reported changes to their institution’s IP funding levels with a median 25.0% decrease in funding (IQR −43.0–1). The IQR describes the middle 50% of the data, when the data is ordered from lowest to highest, from quartile 1 (25th percentile) up to quartile 3 (75th percentile of responses). So, the middle 50% of our respondents reported a 43% decrease in funding (at quartile 1, 25 percentile) up to a 1% increase in funding (quartile 3, or 75% of trauma center respondents), with the median, or middle of the responses, at a 25.0% decrease in funding. These means half of respondents that reported a change in their IP funding had at least a 25.0% decrease in IP funding at their trauma center, with a quarter experiencing a 43% decrease or more in their IP funding.

### Staffing

Prior to the pandemic, respondents reported a median of 1.5 IP staff at their trauma center (IQR 1–4), with only 2 of the 61 centers that responded to this question (3%) reporting no dedicated IP staff. Along with the funding changes, staffing levels were also impacted during the pandemic in 37.7% (*n* = 23) of respondents’ institutions, with a total of 40 staffing changes. For the respondents that specified the staffing change, the most common staffing change was redeployments at 37.5% (*n* = 15/40), followed by lay-off/furlough at 22.5% (*n* = 9/40). A summary of staffing changes is depicted in Table 1.

**Table 1:** Summary of staffing changes during the COVID-19 pandemic reported by respondents (*n* = 40)

Type of staffing change	Redeployment	Layoffs	Staff increase	Terminations	Leave of absence
Percent change	37.5%	22.5%	17.5%	15.0%	7.5%
Median number full-time equivalent (FTE) staff (IQR)	1 (1–1)	0.8 (0.5–1)	1 (0.5–1)	0.6 (0.6–1)	1 (1–1)
Median time (IQR) months^a^	6 (3.75–9)	2.5 (0.5–3.25)	n/a	n/a	3 (3–3)
*Funding source*					
Institution	60.0%	75.0%	28.6%	66.7%	100%
Grant	13.3%	12.5%	71.4%	33.3%	0%
Foundation/donor	6.7%	0%	0%	0%	0%
Don’t know	20.0%	12.5%	0%	0%	0%

^a^Median time of redeployment, leave of absence, or layoff, then return to IP position

### Injury prevention efforts

**Fig. 4:**
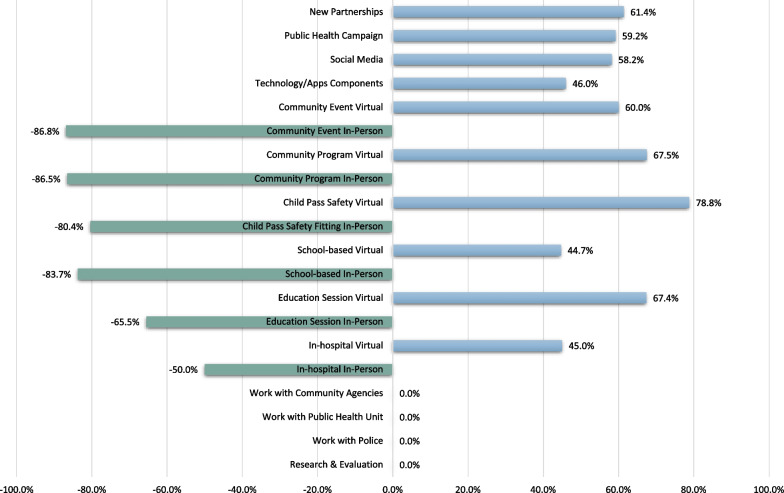
The percentage change in specific types of injury prevention initiatives following the declaration of the COVID-19 pandemic

IP efforts during the COVID-19 pandemic were reported decreased by 63.9% (*n* = 39/61) of respondents, 23.0% (*n* = 14/61) reported no difference of IP efforts and 13.1% (*n* = 8/61) reported an increase in IP efforts (1 response was missing). The percentage change in specific types of IP initiatives is presented in Fig. 4. Any in-person IP initiative decreased from 50.0 to 86.8%, whereas virtual IP programming increased from 45.0 to 78.8%. The majority of respondents (80.0%; *n* = 48/60) stated they will keep a hybrid format of virtual and in-person IP programing, even after public health restrictions were lifted.

### Pivots and innovations

**Fig. 5:**
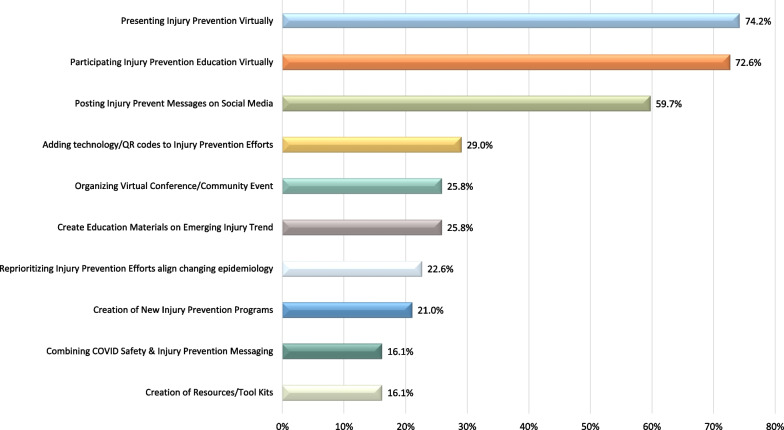
The leading pivots and innovations in injury prevention efforts, as a result of the COVID-19 pandemic and public health restrictions, reported by injury prevention practitioners

With the public health restrictions associated with the COVID-19 pandemic, IP specialists needed to pivot and makes changes to their IP efforts. The leading innovations included presenting and participating in IP education virtually, as well as posing IP messages on social media, at 74.2% (*n* = 46/62), 72.6% (*n* = 45/62) and 59.7% (*n* = 37/62), respectively (Fig. 5).

### Facilitators and barriers

**Fig. 6:**
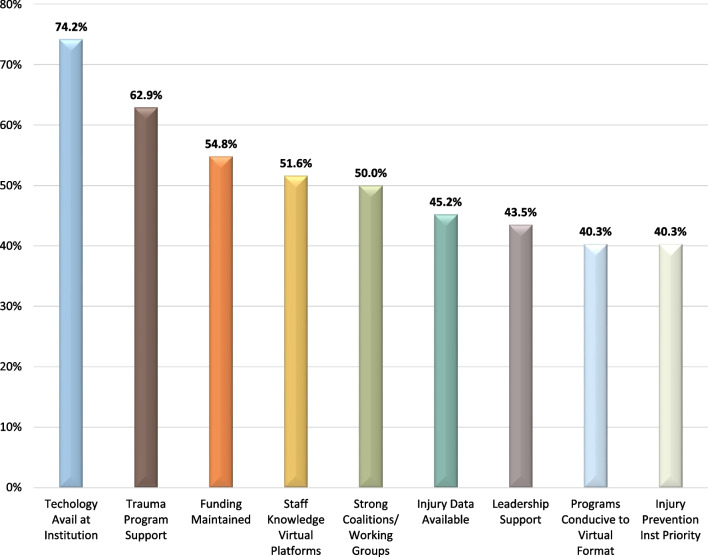
The leading facilitators to pivoting injury prevention efforts during the COVID-19 pandemic

**Fig. 7:**
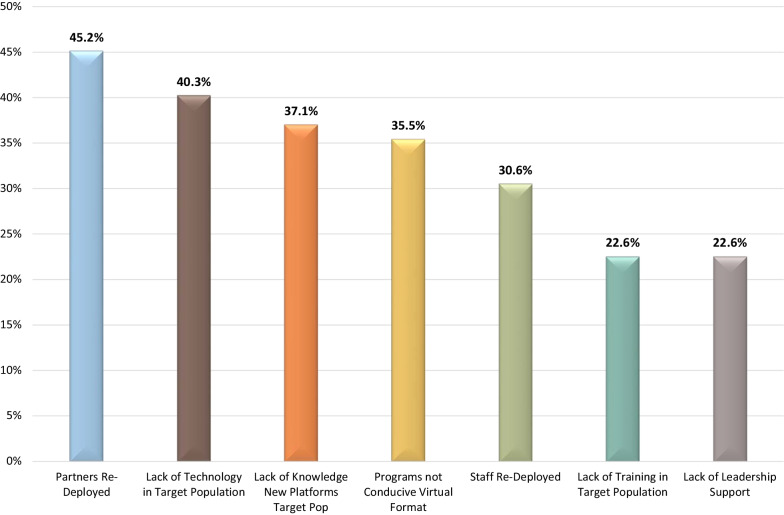
The leading barriers to pivoting injury prevention efforts during the COVID-19 pandemic

Technology (74.2%; *n* = 46/62) with staff knowledge of virtual programs (51.6%; *n* = 32/62), along with support from the trauma program (62.9%; *n* = 39/62) and having IP funding maintained (54.8%; *n* = 34/62) were the leading facilitators to pivoting IP efforts during the pandemic (Fig. 6). Re-deployment of partners (45.2%; *n* = 28/62) and staff (30.6%; *n* = 19/62) and lack of technology (40.3%; *n* = 25/62) and virtual platform knowledge (37.1%; *n* = 23/62) in the target population were important barriers in respondents’ ability to pivot IP efforts (Fig. 7).

### Training

Two-thirds (66.1%; *n* = 41/62) of respondents reported they would benefit from training on designing, implementing, and/or evaluating injury prevention initiatives in a pandemic or times when public health restrictions are in place. The most common training included school or community-based virtual program (73.2%; *n* = 30/41); social media training including posting, the best hashtags to use and social media platforms, as well as the use of analytics (51.2%, *n* = 21/41); followed by evaluation techniques (46.3%; *n* = 19/41).

## Discussion

The WHO declared COVID-19 a world-wide pandemic on March 11, 2020 (World Health Organization 2020). The disease has been devastating, resulting in 1,070,947 COVID-19-related deaths in the US, as of November 9, 2022, making it the third leading cause of death in the US. However, COVID-19–related deaths among children are still rare (Centers for Disease Control and Prevention (CDC) 2022). In fact, the majority of children that contracted COVID-19 were mostly asymptomatic or mildly symptomatic, with a lower risk of hospitalization and severe complications (Nikolopoulou and Maltezou 2022). The true, life-threatening epidemic for children and adolescents remained injuries, with the top three causes of death most recently reported by Centers for Disease Control and Prevention being firearm-related injury, MVC and drug overdoses (Goldstick et al. 2022). With the declaration of the pandemic and implementation of public health directives to stay-at-home and socially distance, youth’s exposure to risk factors for certain injuries was altered, resulting in changes to those injury patterns (Law et al. 2022; Harmon et al. 2021; Keays et al. 2020; Hanson and Pomerantz 2022; Ng et al. 2022).

The leading increases in injuries reported by respondents of our survey reported were intentional mechanisms including assaults, abuse, self-inflicted injury and some firearm-related injuries, which was also reported by other researchers (Abdallah et al. 2021; Rochford et al. 2021). Firearm-related injuries had the largest increases, reported by 75% respondents. This is similar to a study by Collings et al. (2022), which found significant increase in the proportion of firearm injuries affecting children during the COVID-19 pandemic, at rates above what would be expected based on historical patterns. Another study from adult and pediatric trauma centers in Texas found firearm-related injuries almost doubled in 2020 during the pandemic, with over half due to assaults and/or abuse, 36% were accidental shootings, and 10% were self-inflicted (Ng et al. 2022). Hanson and Pomerantz (2022) also found a nearly doubling of the percentage of firearm injuries in 2020 compared to 2019, pre-pandemic. These findings are not surprising given the increased access to firearms that occurred during this time, with an 85% increase in firearm purchases in the United States (Collings et al. 2022). Parents have also made firearms more accessible during the pandemic due to increased civil unrest, threat of home invasion and fear of the unknown (Sokol et al. 2021), and the increased risk in violence and firearm-related behavior due to social and economic stress, as well as social isolation due to the public health restrictions (Rochford et al. 2021). While this was the situation in the United States, the same was not true for Canada. A sub-analysis on firearm-related injuries found five times more American respondents reporting an increase in firearm-related injuries than their Canadian counterparts. This was anticipated, given the differences in gun ownership, gun laws and the issue of gun violence between the United States and Canada, with the CDC reporting a sharp increase in American firearm-related death, mainly from homicides, from 2019 to 2020 (Goldstick et al. 2022).

Assaults and abuse were also on the rise during the pandemic, according to survey respondents, with significantly higher increases reported at American trauma centers compared to Canadian trauma centers. While research results vary on the status of child abuse cases seeking medical attention during the COVID pandemic, two American studies also reported increases in ED visits for child neglect, emotional/psychological abuse, sentinel injuries in infants < 6 months old (Sharma et al. 2021) and physical abuse (Hanson and Pomerantz 2022). Reports failing to find a significant increase in physical abuse during the pandemic may reflect the decrease in contact between children and mandatory reporters, such as teachers, and other adults outside of the home, given the social isolation and move to virtual school platforms (Sharma et al. 2021). The authors strongly suggested the observed decrease in physical abuse is not real, but a reflection of reporting and changes in seeking medical attention, with substantiated decreased ED visits, during the pandemic (Sharma et al. 2021). This aligns with the numerous factors that put unprecedented levels of stress on parents and families due to social isolation from stay-at-home orders, in addition to loss of social supports, job loss or disruptions in employment (Lawson et al. 2020), and increased poverty from an economic downturn (Godinic et al. 2020; Sharma et al. 2021; Matilla-Santander et al. 2021), which may have contributed to child maltreatment. It has been well-established that high stress, as experienced during the pandemic, can increase the risk of child maltreatment (Brown et al. 2020; Rodriguez-JenKins and Marcenko 2014). This was compounded by the closure of schools and halt of recreational activities, access to a structured school environment and participation in extracurricular activities, factors associated with lessening the risk of child maltreatment, was decreased, creating the perfect storm for increased child abuse (Sharma et al. 2021).

The pandemic also exacerbated the illicit drug toxicity crisis, resulting in higher rates of overdose-related events and deaths (Foreman-Mackey et al. 2023). Our results found higher reported rates of unintentional overdose in Canadian trauma centers, as described in other Canadian pandemic research, highlighting the need for ensuring people who use substances are  targeted in future public health emergencies to address this increased overdose risk (Foreman-Mackey et al. 2023).

The increased stressors and changes in daily routines and support systems, along with the widespread social isolation from public health restrictions impacted youth and their mental health during the pandemic, even more than the virus itself (Magson et al. 2021). The resulting increases in violence, overdoses and self-harm injuries emphasized the need to address mental health of children, as well as their parents, as priorities for future IP efforts. Mental health issues are complex with many barriers to at-risk youth being able to access evidence-based mental health services including cost, lack of trained providers, and even transportation issues (Holland et al. 2021b). This makes IP programming that incorporates mental health modalities difficult. Now that school is back to in-person learning in most North American locations, providing school-based prevention and intervention programs that promote social, emotional, and behavioral well-being (Holland et al. 2021b), along with mental health, substance use and violence screening in the ED (Holland et al. 2021a), have been suggested as strategies to help to address mental health issues, provided along with IP initiatives to decrease risk of injury. This recommendation of mental health screening, prevention and services at trauma centers aligns with the new American College of Surgeons (ACS) new *Best Practices Guideline for Screening and Treating Mental Health Disorders and Substance Use and Misuse in the Acute Trauma Patient*, that provides practitioners the tools they need to help identify and treat trauma patients with these needs, as well as comply with the new standard 5.29 that requires Level I and II trauma centers to have a protocol of mental health screening and referral to mental health provider for assessment and treatment for ACS trauma center verification (Fojut 2023).

With the increases in risk factors for both intentional and unintentional injuries, including home and recreational activities, as described in our survey, injury countermeasures are needed more than ever, but two-thirds of our respondents reported a decrease in IP efforts during the pandemic. This supports previous work by Safe States Alliance (Safe States Alliance 2021) that found that the COVID-19 pandemic negatively impacted all areas of injury and violence prevention (IVP) at local and state health departments, as well as hospital-based IVP programs with the IVP workforces called to pause their IVP efforts to help contribute to the COVID-19 response. Our survey also found redeployment of staff and partners to be leading barriers to continuing IP efforts during the pandemic. Unfortunately, our survey did not ask the deployment locations of IP staff, but anecdotally, IP staff at the authors’ trauma centers were deployed to COVID testing centers and vaccination clinics. The reassignment of IP staff to the pandemic response was also reported by Safe States Alliance survey (Safe States Alliance 2021). While the rational to have staff assist in the efforts to combat the pandemic would appear justified to health care administrators, in reality, the more immediate and increasing threat to life, for the children and adolescents in particular, remained trauma, rather than the COVID virus (Safe States Alliance 2021). This questions the logic and impact of these deployment in the community when staff dedicated to the prevention injuries were no longer able to do their interventions and prevention programs, despite the increasing need (Safe States Alliance 2021). This should be taken into consideration if a future global public health crisis arises. In addition, the Safe States Alliance study (Safe States Alliance 2021) reported staffing and funding to also be negatively impacted at hospital-based IVP programs by the pandemic by 50% and 30%, respectively. This corroborated our survey findings, with 38% staffing and 15% funding changes reported, but to an even higher level.

In response to social distancing and the limitations on large gatherings, IP practitioners reported pivoting to a virtual environment for their programming as the main response to the pandemic public health restrictions. While this allowed many trauma centers to continue to reach youth, it also created access issues for some children and adolescents. Leading barriers reported in our survey were the lack of technology and lack of knowledge of new platforms in the target population. Lack of access to or difficulty upgrading technology was also identified as a barrier for 43% respondents in the Safe States Alliance survey (Safe States Alliance 2021), as well as difficulty with new technology software and platforms including Zoom or Microsoft Teams at 36%. This was similar to 40% and 37% of our respondents reporting these barriers, respectively.

COVID-19 amplified the social inequities and disparities that existed between groups of youth with differing social determinants of health (Frohlich et al. 2022), and the same was found with the delivery of IP initiatives. If members of the target population did not have access to computers or Wi-Fi, or lacked knowledge of how to utilize the platforms used for IP education or virtual programs, then they were not able to participate in the initiative. This is of grave concern, given children with access issues to virtual IP programs were likely the population at greatest risk for injury. This deepening of existing social inequities for youth by COVID-19 has been reported for other types of education (Frohlich et al. 2022). In essence, a “digital divide” was created by the pandemic as a result of access to technology and this was not experienced equally across all social and racial groups of youth, impacting some populations more than others (Safe States Alliance 2021). Identifying as Indigenous, living in a rural community, having a low income and living with disabilities were risk factors reported to be associated with access issues (Safe States Alliance 2021). Programming for youth with these risk factors will need to be taken into account and access issues addressed by IP practitioners as we move forward, as the majority of respondents reported plans to have a hybrid of virtual and in-person programming for their future IP efforts. While not specifically asked in our study, it has been reported that IP staff have found, after the initial challenges with moving to a virtual or hybrid model, successes with moving programs and services online including more efficient and well-attended meetings within programs and meetings with partners (Safe States Alliance 2021). Access was improved for some participants (Safe States Alliance 2021), but this will need to be reviewed within the target audience to ensure all participants have equal access to IP initiatives to reduce their risk of injury.

On the positive side, IP efforts were able to continue at the same or even an increased rate by over a third of our respondents. Important facilitators to carry on with IP work and pivot to a new method were technology at the institution and having a strong support system from the Trauma Program and leadership, with the recognition that IP was still an institutional priority and funding was maintained. This needs to be kept in mind, as we now move from COVID-19 to other infectious disease resurgences and respiratory ailments that have manifested during the COVID-19 pandemic for children (Billard et al. 2022). As IP practitioners, we need to continue to build strong relationships with institutional and government leadership for continued funding and support, because whatever infectious disease or pandemic comes next, for the time being, injury remains the most important epidemic killing our kids.

Our study does have some limitations. As a survey, it is subject to the potential biases associated with all self-report research including providing socially desirable responses and recall bias (Althubaiti 2016). For example, our survey asked respondents about injury trends during the pandemic based on their review of the data, but did not collect the injury data. The type and amount of data reviewed, along with their accurate recall of it, cannot be quantified and may have impacted their perception and reporting of injury trends during the pandemic. This could result in selective recall bias (Althubaiti 2016). However, the impact of this is likely minimal given the results of our survey on injury trends have been found to align with studies based on ED or Trauma Registry data (Harmon et al. 2021; Keays et al. 2020; Hanson and Pomerantz 2022; Ng et al. 2022), providing an external validation of our survey data with administrative data (Althubaiti 2016). Another limitation is the inability to calculate a response rate due to the use of snowball sampling. Despite the lack of a denominator, we know that there were 62 respondents, all from different trauma centers, so there was no duplication in responses from one or more trauma centers, thereby eliminating any bias that would result from overrepresentation responses from a trauma center. A third limitation is the extent of the generalizability of the results. While the study was international, it was primarily focused on North America, with the sampling frame derived from North American-based trauma and injury prevention associations. Given 82% of the responses were from the United States, it is most representative of American trauma centers, so that needs to be considered when generalizing these results. Finally, this survey was distributed in the fall of 2021, and the results reflect that time period, a year and a half into the COVID-19 pandemic. Injury trends and IP practices may differ now and may not be generalizable to the current situation, without the same level of public health restrictions currently in place. That being said, other infectious diseases and pandemics may be on the horizon and we all can learn from our COVID-19 experience to improve IP efforts in future to help keep youth safe.

## Conclusions

Nearly two-thirds of trauma centers decreased IP efforts during the pandemic due to staffing and funding reductions. The leading reported increased injury mechanisms were intentional, so further violence and self-harm screening and prevention is needed at trauma centers, along with support for mental health. While trauma centers successfully pivoted by using technology and going virtual, access issues in the target population were a barrier resulting in health inequities.

### Supplementary Information

**Additional file** **1:** Copy of Final Survey: Impact of the COVID-19 Pandemic on Injury and its Prevention.

## Data Availability

The datasets generated and analyzed during the current study are not publicly available due to potentially identifying information but are available from the corresponding author on reasonable request.

## Abbreviations

ACS: American College of Surgeons
CROSS: Consensus-Based Checklist for Reporting of Survey Studies
ED: Emergency department
FTE: Full-time equivalent
HSREB: Health Sciences Research Ethics Board
IBM®: International Business Machines
IFCK: Injury Free Coalition for Kids®
IQR: Interquartile range
IP: Injury prevention
IVP: Injury and violence prevention
MVC: Motor vehicle collisions
PTS: Pediatric Trauma Society
REDCap: Research Electronic Data Capture
SPSS®: Statistical Package for the Social Sciences
TAC: Trauma Association of Canada
TC: Trauma centers
US: United States
WHO: World Health Organization
