# Supplementary material for: Pivoting injury prevention efforts during a pandemic: results of an international survey
Source: Inj Epidemiol. 2023 Nov 16;10(Suppl 1):59. doi: 10.1186/s40621-023-00472-3 (PMC10652424; doi:10.1186/s40621-023-00472-3)
Supplement: Supplementary file 1 — Additional file 1: Copy of Final Survey: Impact of the COVID-19 Pandemic on Injury and its Prevention. [file 40621_2023_472_MOESM1_ESM.pdf]

Impact of the COVID-19 Pandemic on Injury and its Prevention

If you perform injury prevention activities as part of the responsibilities of your job, please complete the survey below.

If your job does not involve injury prevention activities, please feel free to forward this invitation with the survey link on to the Injury Prevention Coordinator or Specialist at your institution, so your institution's experience during the COVID-19 pandemic can be represented.

Thank you!

Letter of Information
Impact of the COVID-19 Pandemic on Injury and its Prevention
Principal Investigator: Tanya Charyk Stewart, MSc
Study Team: Jason Gilliland, PhD, Andrew Clark, PhD, Purnima Unni, MPH, Holly R. Hanson, MD, MS, & Douglas D. Fraser, MD, PhD

The COVID-19 pandemic, along with the public health measures to “flatten the curve” including social distancing, staying home, working from home, the closure of non-essential work places and schools, as well as closing recreation spaces and stopping organized sport, has impacted not only injury risk factors and injury patterns, but also prevention efforts to reduce injury. Our injury research team is conducting a survey to better understand how the COVID-19 pandemic and associated public health directives have impacted injury prevention efforts in both Canada and the US. The survey will be sent to approximately 1,200 potential participants. The way we all do injury prevention activities has changed as a result of the pandemic, and we would like to learn how prevention efforts have been altered, as well as innovations arising from restrictions.

This survey is anonymous and you will not be identified. It is on the secure, confidential web application REDCap, which does not track IP addresses, e-mail addresses, or any other identifying information. All information you provide will remain confidential, in password protected electronic files on encrypted computers, accessible only to the principal investigator, not external study team members, and will be used only for the purposes of this research. Western’s Research Ethics Board may require access to your study-related records to monitor the conduct of the research and Lawson’s Quality Assurance and Education Program may audit this research study for quality assurance purposes, so they have access to all study data, which will be stored for the required period of 15 years. You may refuse to participate, refuse to answer any of the questions, or withdraw at any time by simply closing the survey or not submitting it. However, once the survey has been submitted it cannot be withdrawn due to the nature of the survey platform. Participation in this survey is voluntary and does not involve any risks or harms. If you complete and submit this survey, it will be understood that you have consented to participate. Benefits to your participating is gaining information to better understand how the COVID-19 pandemic has altered injury prevention efforts, to help design and implement future prevention initiatives for the reduction of injuries.

If you have any questions about this study, please contact the Principal Investigator, Tanya Charyk Stewart, Injury Epidemiologist at tanya.charykstewart@lhsc.on.ca. If you have any questions regarding your rights as a research participant or the conduct of this study, you may contact the Office of Research Ethics at Western University at ethics@uwo.ca (519) 661-3036, 1-844-720-9816 and/or the Patient Relations Office at London Health Sciences Centre at (519) 685-8500 ext. 52036. This letter is attached and can be downloaded and saved for your records. Thank you for considering participation in this study. Again, continuing on to the survey is your consent to participant.

Tanya Charyk Stewart, MSc
Principal Investigator; Injury Epidemiologist & Data Specialist, LHSC
Adjunct Research Professor, Dept of Paediatrics; Dept of Pathology & Laboratory Medicine, Schulich School of Medicine & Dentistry
Associate Scientist, Lawson Health Research Institute

Western Research Park, Convergence Centre, 999 Collip Circle, Rm LL02, London, Ontario, Canada N6G 0J3

Email: tanya.charykstewart@lhsc.on.ca

[Attachment: "Letter of Information COVID Injury and its Prevention.pdf"]

I. Injury Prevention Initiatives

What are the age groups your injury prevention efforts target?

Check all that apply.

- ☐ Infants (< 1 year)
- ☐ Toddlers (1-4 years)
- ☐ School age (5-9 years)
- ☐ Early Adolescence (10-15 years)
- ☐ Late Adolescence (16-17 years)
- ☐ Young Adults (18-24 years)
- ☐ Adults (25-54 years)
- ☐ Seniors (>= 55 years)
- ☐ All ages

(Check all that apply)

Since the start of the COVID-19 pandemic, the overall level of your institution's injury prevention efforts has:

- ◯ Extremely increased
- ◯ Moderately increased
- ◯ Slightly increased
- ◯ Remained the same
- ◯ Slightly decreased
- ◯ Moderately decreased
- ◯ Extremely decreased

II. Injury Data

Since the start of the COVID-19 pandemic, has injury data been reviewed to keep the Injury Prevention team informed of current injury epidemiology?

- ◯ Yes
- ◯ No

Please check all data sources that have been reviewed:

- ☐ Emergency Department data
- ☐ Hospital In-Patient Data
- ☐ Trauma Registry Data
- ☐ Coroner's/Death Data
- ☐ Injury-specific databases (i.e., MVC data, abuse registries, etc.)
- ☐ Other injury data, please specify: ______

(Check all sources reviewed by someone on IP &/or Trauma Program Team)

For each of the following injury mechanisms, please specify any trends in the number of cases seen at your institution since the start of the COVID-19 pandemic.

If you haven't reviewed or been informed of changes in volumes to one of these mechanism of injury, please specify "Don't know".

	Extremely decreased	Moderately decreased	Slightly decreased	Remained the same	Slightly increased	Moderately increased	Extremely increased	Don't know
MVC Occupant Injuries	◯	◯	◯	◯	◯	◯	◯	◯
Motorcycle Injuries	◯	◯	◯	◯	◯	◯	◯	◯
Pedestrian Injuries	◯	◯	◯	◯	◯	◯	◯	◯
Cyclist Injuries	◯	◯	◯	◯	◯	◯	◯	◯
ATV Injuries	◯	◯	◯	◯	◯	◯	◯	◯
Home Injuries	◯	◯	◯	◯	◯	◯	◯	◯

Falls from windows	◯	◯	◯	◯	◯	◯	◯	◯
Unintentional poisonings/ingestions	◯	◯	◯	◯	◯	◯	◯	◯
Unintentional overdoses	◯	◯	◯	◯	◯	◯	◯	◯
Intentional poisonings/overdoses	◯	◯	◯	◯	◯	◯	◯	◯
Abuse/Non-Accidental Trauma	◯	◯	◯	◯	◯	◯	◯	◯
Assaults	◯	◯	◯	◯	◯	◯	◯	◯
Self-inflicted Injury/Suicide	◯	◯	◯	◯	◯	◯	◯	◯
GSW	◯	◯	◯	◯	◯	◯	◯	◯
Tobogganing/Sledding Injuries	◯	◯	◯	◯	◯	◯	◯	◯
Playground Injuries	◯	◯	◯	◯	◯	◯	◯	◯
Trampoline Injuries	◯	◯	◯	◯	◯	◯	◯	◯
Drownings	◯	◯	◯	◯	◯	◯	◯	◯
Sports Injuries	◯	◯	◯	◯	◯	◯	◯	◯
Animal bites	◯	◯	◯	◯	◯	◯	◯	◯
Farm Injuries	◯	◯	◯	◯	◯	◯	◯	◯
Other Injuries 1, please specify ______	◯	◯	◯	◯	◯	◯	◯	◯
Other Injuries 2, please specify ______	◯	◯	◯	◯	◯	◯	◯	◯

Based on the injury data, are there new injury prevention programs/interventions/campaigns you plan on implementing as a result of the COVID-19 Pandemic and changes in injury epidemiology?

◯ Yes, please specify ______
◯ No

III. Staffing

Prior to the COVID-19 pandemic, how many Full Time Equivalent (FTE) Injury Prevention Staff did your institution have?

____
(Note: 1 for full-time staff, 0.2 for each day worked for part-time staff)

Did your institution have any injury prevention staffing changes due to the COVID-19 pandemic?

◯ Yes
◯ No

Please enter up to 5 staffing changes (decreases or increases) since the pandemic, along with the full-time equivalent (FTE) (i.e., 1 for full-time staff, 0.2 for each day worked for part-time staff).

For redeployment, temporary lay-off/furlough or leave of absence (LOA), please enter the length of time.

If a position was funded by separate sources, please enter on separate rows, specifying the FTE by each funding source.

Staffing Change FTE

1 day=0.2 FTE

Length of Redeployment, Lay-off or LOA How was position funded?

______ ______ ______ ______ ______
______ ______ ______ ______ ______
______ ______ ______ ______ ______
______ ______ ______ ______ ______
______ ______ ______ ______ ______

IV. Injury Prevention Funding & Priority

Do you know how injury prevention was funded at your institution prior to the COVID-19 pandemic?

◯ Yes
◯ No
◯ Don't know

Please enter the % of injury prevention funding, by source, prior to the pandemic.

Type of Funding Prior to COVID Pandemic (Ensure % from all 3 sources added together = 100%)
Institution ______
Grants ______
Foundation/Donor ______

Has your institution had any changes in injury prevention funding since the start of the COVID-19 pandemic?

◯ Yes
◯ No
◯ Don't know

If yes, please enter the changes to the injury prevention funding, by source, since the start of the pandemic, along with the impact of any decreases, or increases, in funding.

Type of Funding % Change in Funding Since COVID Pandemic Impact of Funding Changes
Institution ______ ______
Grants ______ ______
Foundation/Donor ______ ______

Please select the response that best describes the level of importance of Injury Prevention as an institutional priority, since the declaration of the COVID-19 pandemic.

	Extremely less important priority	Moderately less important priority	Slightly less important priority	No change in level of importance as a priority	Slightly more important priority	Moderately more important priority	Extremely more important priority	Don't know
Since the start of the pandemic, how do you feel your institution views injury prevention in terms of an institutional priority:	◯	◯	◯	◯	◯	◯	◯	◯

V. Changes in Injury Prevention Efforts Due to the Pandemic

Please specify any innovations with your injury prevention efforts since the start of the COVID-19 Pandemic.

Check all that apply & provide further details or links, if you are able.
(Check all that apply)

☐ Presenting Injury Prevention Education virtually via Zoom/WebEx/other platform
☐ Participating in Injury Prevention Education virtually via Zoom/WebEx/other platform
☐ Posting Injury Prevention Messages on Social Media, specify platforms: ______
☐ Development of public health social media/social marketing campaigns, please specify or provide link: ______
☐ Combining COVID-19 prevention/safety with Injury Prevention/safety messaging, please specify or provide link: ______
☐ Organized a virtual conference/community event, please specify: ______
☐ Creation of new Injury Prevention Programs, please specify: ______
☐ Creation of educational materials on emerging injury trends, please specify: ______
☐ Creation of resources/tool kits, please specify: ______
☐ Adding technology/apps/digital-based components to Injury Prevention efforts (i.e., QR codes, text reminders, online surveys, etc.), please specify: ______
☐ Reprioritizing injury prevention efforts to align with changing pediatric trauma epidemiology (i.e., home injuries, abuse, etc.), please specify new priority: ______
☐ Other innovations, please specify: ______
☐ No innovations

Please identify the level and direction of change in the following types of injury prevention efforts at your institution, since the start of the COVID-19 pandemic.

If your institution doesn't have a specific type of program or partnership, please respond "N/A".

	Extremely decreased	Moderately decreased	Slightly decreased	Remained the same	Slightly increased	Moderately increased	Extremely increased	N/A
In-hospital prevention programs (In-person)	○	○	○	○	○	○	○	○
In-hospital prevention programs (Virtual)	○	○	○	○	○	○	○	○
Injury prevention education sessions (In-person)	○	○	○	○	○	○	○	○
Injury prevention education sessions (Virtual)	○	○	○	○	○	○	○	○
School-based prevention programs (In-person)	○	○	○	○	○	○	○	○
School-based prevention programs (Virtual)	○	○	○	○	○	○	○	○
Child passenger safety fitting station (In-person)	○	○	○	○	○	○	○	○
Child passenger safety fitting station (Virtual)	○	○	○	○	○	○	○	○
Other community-based prevention programs (In-person)	○	○	○	○	○	○	○	○

Other community-based prevention programs (Virtual)	○	○	○	○	○	○	○	○
Community events (In-person)	○	○	○	○	○	○	○	○
Community events (Virtual)	○	○	○	○	○	○	○	○
Social media use (i.e., Twitter, TikTok, Instagram, Snapchat, YouTube, etc.)	○	○	○	○	○	○	○	○
Public health social media/social marketing campaigns	○	○	○	○	○	○	○	○
Technology/apps/digital-based components (i.e., QR codes) to programs	○	○	○	○	○	○	○	○
Injury prevention research & evaluation	○	○	○	○	○	○	○	○
Work with police forces	○	○	○	○	○	○	○	○
Work with public health units	○	○	○	○	○	○	○	○
Work with other community agencies, specify: ______	○	○	○	○	○	○	○	○
Developed new partnerships	○	○	○	○	○	○	○	○
Other, specify: ______	○	○	○	○	○	○	○	○

As public health restrictions are lifted and we move forward from the COVID-19 pandemic, what format do you feel most injury prevention programs at your institution will take in the future?

○ Virtual
○ Hybrid of virtual & in-person
○ In-person

VI. Facilitators & Barriers to Pivoting During the Pandemic

Please specify the facilitators allowed you to successfully pivot your injury prevention efforts during the COVID-19 pandemic
(Check all that apply)

- ☐ Technology available at institution
- ☐ Technology available in target population
- ☐ Technical support available
- ☐ Knowledge on virtual platforms/technology in staff
- ☐ Knowledge on virtual platforms/technology in target population
- ☐ Training available for staff
- ☐ Training available for target population
- ☐ Resources/tool kits available
- ☐ Strong working groups/coalitions/networks
- ☐ Staff not re-deployed
- ☐ Staff increased
- ☐ Partners not re-deployed
- ☐ Leadership support
- ☐ Trauma Program support
- ☐ Injury prevention remained institutional priority
- ☐ Injury data availability
- ☐ Funding maintained
- ☐ Funding increased
- ☐ Programs conducive to be transitioned to virtual format
- ☐ Other facilitators, please specify ______
- ☐ No facilitators/None of the above

Please specify the barriers that hindered the pivot of your injury prevention efforts during the COVID-19 pandemic
(Check all that apply)

- ☐ Lack of technology at institution
- ☐ Lack of technology in target population
- ☐ Lack of technical support
- ☐ Lack of knowledge on new platforms/technology in staff
- ☐ Lack of knowledge on new platforms/technology in target population
- ☐ Lack of training available for staff
- ☐ Lack of training available for target population
- ☐ Lack of resources/tool kits
- ☐ Staff re-deployed
- ☐ Partners re-deployed/no longer available
- ☐ Lack of working groups/coalitions/networks
- ☐ Lack of leadership support
- ☐ Lack of Trauma Program support
- ☐ Injury prevention no longer an institutional priority
- ☐ Lack of injury data
- ☐ Lack of funding
- ☐ Programs not conducive to be transitioned to virtual
- ☐ Other barriers, please specify ______
- ☐ No barriers/None of the above

VII. Education & Training

Overall, since the start of the COVID-19 pandemic, do you feel your own injury prevention education/professional development (i.e., conferences, webinars) has:

- ◯ Extremely increased
- ◯ Moderately increased
- ◯ Slightly increased
- ◯ Remained the same
- ◯ Slightly decreased
- ◯ Moderately decreased
- ◯ Extremely decreased

Do you feel you would benefit from training on designing, implementing &/or evaluating injury prevention initiatives during a pandemic?

- ◯ Yes
- ◯ No

Specify the type of training you would like.

Check all that apply.
(Check all that apply)

☐ School/community-based virtual programming
☐ Ensuring in-person programs are public health compliant (i.e., sanitizing, social distancing, masks, etc.)
☐ Social media training (i.e., posting, best platforms to use, hashtags, analytics, etc.)
☐ Developing a public health social media/social marketing campaigns
☐ Developing resources/tool kits
☐ How to combine COVID-19 prevention/safety with Injury Prevention/safety messaging
☐ Presenting on Zoom, WebEx, or other virtual platforms
☐ Organizing a virtual conference/community event
☐ Utilizing team-based software for team meetings and collaborations (i.e., MS Teams, Trello, etc.)
☐ Utilizing technology/apps/digital-based components (i.e., QR codes) into Injury Prevention initiatives
☐ How to utilize data/changes in injury epidemiology to drive Injury Prevention Programming
☐ Techniques to evaluate a program during a pandemic
☐ Other, please specify: ______

Would you like to participate in any educational sessions on injury prevention programming ideas, revisions &/or innovations as a result of the pandemic?

○ Yes
○ No

Please specify any suggested topic areas:

____

If you have an injury prevention programming idea, revision or innovation that you implemented due to the pandemic that you would be willing to present on, please provide your email address to be contacted by our team:

____
(Valid email address format only i.e., joe@user.com)

Do you have any other comments related to injury prevention and how it has changed since the COVID-19 pandemic? ______

○ Yes
○ No

VIII. Demographics

How many years have you been working in injury prevention?

____
(years)

What best describes your role?

○ Injury Prevention Coordinator/Specialist
○ Trauma Program Coordinator/Manager
○ RN
○ Nurse Educator
○ Clinical Nurse Specialist/Nurse Practitioner
○ Physician Assistant
○ Social Worker
○ Researcher
○ Resident/Fellow
○ Attending Physician
○ Trauma Medical Director
○ Other, please specify ______

Please specify the trauma association(s) you belong to:	☐ American Association for the Surgery of Trauma (AAST)<br>☐ Australasian Trauma Society (ATS)<br>☐ Eastern Association for the Surgery of Trauma (EAST)<br>☐ Injury Free Coalition for Kids (IFCK)<br>☐ Pediatric Trauma Society (PTS)<br>☐ Society of Trauma Nurses (STN)<br>☐ Trauma Association of Canada (TAC)<br>☐ Other, please specify: ______<br>(Check all that apply)
How do you identify your gender?	◯ Male<br>◯ Female<br>◯ Non-binary<br>◯ Prefer to self-describe, please specify: ______<br>◯ Prefer not to answer
What is your age?	______________________________<br>(years)
Name of your institution:	______________________________<br>(To group responses by institution ~ Will not be reported)
Your institution type:	◯ Pediatric Trauma Centre<br>◯ Adult Trauma Centre<br>◯ Combined Adult & Pediatric Trauma Centre<br>◯ Public Health<br>◯ Government Agency<br>◯ Injury Prevention Organization<br>◯ Other, please specify: ______
Level of your Pediatric Trauma Centre:	◯ Level I<br>◯ Level II<br>◯ Level III<br>◯ Level IV<br>◯ Level V
Level of your Adult Trauma Centre:	◯ Level I<br>◯ Level II<br>◯ Level III<br>◯ Level IV<br>◯ Level V
Please specify the country you work in:	◯ Canada<br>◯ United States

Select your province:

- ◯ AB - Alberta
- ◯ BC - British Columbia
- ◯ MB - Manitoba
- ◯ NB - New Brunswick
- ◯ NL - Newfoundland & Labrador
- ◯ NS - Nova Scotia
- ◯ NT - Northwest Territories
- ◯ NU - Nunavut
- ◯ ON - Ontario
- ◯ PE - Prince Edward Island
- ◯ QC - Quebec
- ◯ SK - Saskatchewan
- ◯ YT - Yukon

Select your state:

- ◯ AL - Alabama
- ◯ AK - Alaska
- ◯ AZ - Arizona
- ◯ AR - Arkansas
- ◯ CA - California
- ◯ CO - Colorado
- ◯ CT - Connecticut
- ◯ DE - Delaware
- ◯ DC - District of Columbia
- ◯ FL - Florida
- ◯ GA -Georgia
- ◯ HI - Hawaii
- ◯ ID - Idaho
- ◯ IL - Illinois
- ◯ IN - Indiana
- ◯ IA - Iowa
- ◯ KS - Kansas
- ◯ KY - Kentucky
- ◯ LA - Louisiana
- ◯ ME - Maine
- ◯ MD - Maryland
- ◯ MA - Massachusetts
- ◯ MI - Michigan
- ◯ MN - Minnesota
- ◯ MS - Mississippi
- ◯ MO - Missouri
- ◯ MT - Montana
- ◯ NE - Nebraska
- ◯ NV - Nevada
- ◯ NH - New Hampshire
- ◯ NJ - New Jersey
- ◯ NM - New Mexico
- ◯ NY - New York
- ◯ NC - North Carolina
- ◯ ND - North Dakota
- ◯ OH - Ohio
- ◯ OK - Oklahoma
- ◯ OR - Oregon
- ◯ PA - Pennsylvania
- ◯ RI - Rhode Island
- ◯ SC - South Carolina
- ◯ SD - South Dakota
- ◯ TN - Tennessee
- ◯ TX - Texas
- ◯ UT - Utah
- ◯ VT - Vermont
- ◯ VA - Virginia
- ◯ WA - Washington
- ◯ WV - West Virginia
- ◯ WI - Wisconsin
- ◯ WY - Wyoming

Please specify your city:

____
